# Systemic Stressors and Retinal Microvascular Alterations in People Without Diabetes: The Kailuan Eye Study

**DOI:** 10.1167/iovs.62.2.20

**Published:** 2021-02-17

**Authors:** Wenjia Zhou, Jingyan Yang, Qian Wang, Yaxing Wang, Yanni Yan, Shouling Wu, Shuohua Chen, Wenbin Wei

**Affiliations:** 1 Beijing Tongren Eye Center, Beijing Tongren Hospital, Beijing Ophthalmology and Visual Science Key Lab, Beijing Key Laboratory of Intraocular Tumor Diagnosis and Treatment, Capital Medical University, Beijing, China; 2 Beijing Institute of Ophthalmology, Beijing Key Laboratory of Ophthalmology and Visual Sciences, Beijing Tongren Eye Center, Beijing Tongren Hospital, Capital Medical University; Beijing, China; 3 Department of Cardiology, Kailuan General Hospital, Tangshan, China; 4 Health Care Center, Kailuan Group, Tangshan, China

**Keywords:** fasting plasma glucose, optical coherence tomographic angiography (OCTA), capillary density, retinal microvasculature, atherosclerotic cardiovascular disease (ASCVD)

## Abstract

**Purpose:**

The purpose of this study was to determine systemic stressors, including fasting plasma glucose (FPG), and other major atherosclerotic cardiovascular disease (ASCVD) risk factors of the retinal microvasculature in people without diabetes.

**Methods:**

The Kailuan Eye Study enrolled applicants from the community-based longitudinal Kailuan Study. Applicants underwent optical coherence tomographic angiography (OCTA) and systemic examinations. Both the macula and optic disc were screened, whereas superficial capillary plexus (SCP), deep capillary plexus (DCP), foveal vessel density in the 300 µm ring (FD-300), and radial peripapillary capillaries (RPCs) density were measured in the study.

**Results:**

This study included 353 eligible applicants (mean age = 49.86 ± 11.41 years; 47% men; FPG =5.32 ± 1.19 mmol/L). Lower DCP density was associated with elder age (*P* = 0.001), male gender (*P* < 0.001), and higher FPG (*P* = 0.008). Male gender (*P* < 0.001), axial length (*P* < 0.001), and FPG (*P* = 0.029) were inversely associated with RPC density. Meanwhile, a higher FPG concentration was significantly correlated with lower DCP density (*P* = 0.006) and higher intraocular pressure (*P* = 0.006), after adjusting mean arterial blood pressure (*P* = 0.001) and sex (*P* = 0.042).

**Conclusions:**

DCP density showed a significantly negative correlation with FPG concentration in people without diabetes. These data suggest hyperglycemia could cause early retinal capillary alterations in patients without clinical signs of retinopathy and indicate the potential clinical applications of routine OCTA may be beneficial to screen for subclinical microvasculature and monitor patients with high risks of ASCVD.

Atherosclerotic cardiovascular disease (ASCVD) remains the leading cause of morbidity and mortality in patients with diabetes in China and worldwide.^1^^,^^2^ The risk factors of diabetes, including hypertension, hypercholesterolemia, and adverse health behavior (such as smoking, poor diet, and elevated body mass index), also contribute to ASCVD burden and mortality*.* Extensive studies have been done to reduce the burden of ASCVD, but there still remains insufficient preventative interventions and difficulty maintaining ideal cardiovascular health conditions.^1^ According to the updated statistics, approximately 451 million people (age 18–99 years) live with diabetes, and this is expected to increase to 693 million by 2045 worldwide. Almost half of patients with diabetes (49.7%) have not been diagnosed, and an estimated 374 million people live with impaired glucose tolerance (IGT).^3^ The global prevalence of hypertension is estimated to be 1.13 billion worldwide, and the overall prevalence of hypertension with a global age standardized is 24% and 20% in men and women, respectively.^4^ Persistent hyperglycemia or hypertension can cause generalized vascular damage to the heart, eyes, kidneys, and nerve cells, leading to various complications.^5^ Because the only vessels visible in real time through noninvasive imaging tools are in the retina, the relationships between systemic stressors and the retinal vasculature are of great significance. Wong's group^6^^,^^7^ reported retinal vascular tortuosity and caliber were related to ASCVD risk factors. However, some systemic parameters are considered to be protective against retinopathy, including elevated serum bilirubin, was reported to be significantly correlated with lower diabetic retinopathy (DR) prevalence in patients with diabetes mellitus (DM) or IGT.^8^

Optical coherence tomographic angiography (OCTA) is a noninvasive technique that allows analysis of retinal vasculature and morphology. Unlike other traditional ophthalmic imaging tools, OCTA could automatically segment specific capillary plexus layers and visualize the structure,^9^ such as retinal thickness and capillary perfusion. In addition, early-stage impairments might be rarely seen on color photographs, yet OCTA scans are crucial for those in the regional and capillary level lesions. Massive studies have valued OCTA as a novel tool to evaluate the microvasculature in systemic disorders. Patients with hypertension and diabetes were reported to have an enlarged foveal avascular zone (FAZ), and decreased retinal capillary density.^10^^–^^12^ In addition, alterations in FD-300 density, FAZ perimeter and acircularity index were found in patients with type I diabetes.^13^ O'Bryhim's group^14^ reported inner foveal thickness was decreased in the Alzheimer's biomarker-positive group compared with healthy controls. However, recent findings of correlation between multiple systemic risk factors and retinal microvascular impairment from a Chinese population-based cohort are limited. In this study, we aimed to describe the prognostic significance of OCTA microvascular parameters and their associations with age, gender, fasting plasma glucose (FPG), blood pressure, and other major ASCVD risk factors in patients without diagnosed diabetes, or showing any clinical evidence of DR, to determine whether the retinal microvasculature may be a useful indicator of subclinical damage in people with high risk of diabetes or ASCVD. Furthermore, we hypothesize the implications of rapid ocular examinations could be applied as surrogates for the coronary and systemic circulation, and may provide essential information to stratify patients with high risk of severe ASCVD events.

## Methods

### Study Population

The Kailuan Eye Study is a cross-sectional study that included Chinese participants of a longitudinal community-based cohort study. At baseline in year 2006, 101,510 working and retired employees aged from 18 and 98 years were included in the cohort and were examined repeatedly and prospectively at 2-year intervals.^15^ Based on the data of the 2010 Chinese National Census, 14,440 individuals were randomly selected and agreed to participate the Kailuan Eye Study out of the Kailuan cohort. All participants received ophthalmic examination and standardized interviews and laboratory investigations they had on each interval, including the major ASCVD parameters. From March 2017 to June 2017, 390 eligible applicants were randomly recruited and underwent OCTA examination additionally. Participants with physician-diagnosed DM or clinical evidence of DR on digital fundus photographs were excluded from the present study. DR was assessed in a masked manner following the Early Treatment of Diabetic Retinopathy Study (ETDRS) criteria without awareness of ocular or systemic information of the study participants.^16^ Other exclusion criteria were the history of ASCVDs, including myocardial infarction and stroke, ocular diseases (except for age-related cataract), and previous ocular surgeries (except for cataract or Lasik surgeries). Scans that had low signal strength (OCTA Scan Quality Index < 6) or blurred images were also excluded from the study. Participants with myopia, incipient cataract, or pseudophakia that did not interfere with the OCTA imaging qualities were not excluded. The Kailuan Eye Study was conducted adhered to the Declaration of Helsinki and approved by the Medical Ethics Committee of the Beijing Tongren Hospital. Written informed consent was obtained from each participant.

### Assessment of ASCVD Risk Factors and Other Systemic Variables

All participants had undergone standardized and systemic examinations to collect clinical data and personal information. Trained interviewers administered questionnaires individually to collect demographic information, sociodemographic characteristics, lifestyle patterns (smoking, alcohol, and exercise), and self-reported medical history (diabetes, hypertension, dyslipidemia, cardiovascular disease, thyroid diseases, stroke, family history, current medication intake, etc.). Body mass index (BMI) was calculated by using the formula of weight (kg)/height (m^2^) and the waist-hip ratio was then calculated. The blood pressure and heart rate were assessed by professional doctors with a standardized mercury sphygmomanometer when participants had been in the sitting position for at least 5 minutes. Two measurements of blood pressure were recorded at a 5-minute interval and the average was recorded for analysis. Mean arterial blood pressure (MABP) was then calculated as two thirds of the diastolic plus one third of the systolic blood pressure. After an overnight fasting condition, blood samples were collected from all subjects. FPG, high-density lipoprotein cholesterol (HDL-C), low-density lipoprotein cholesterol (LDL-C), triglyceride (TG), total cholesterol (TC), total protein, albumin, uric acid, hypersensitive C-reactive protein (H-CRP), glutamate pyruvate transaminase (GPT), total serum bilirubin, and serum creatinine were tested at the clinical laboratory of the Kailuan General Hospital.

### Ocular Examinations and Imaging

**Figure 1.**
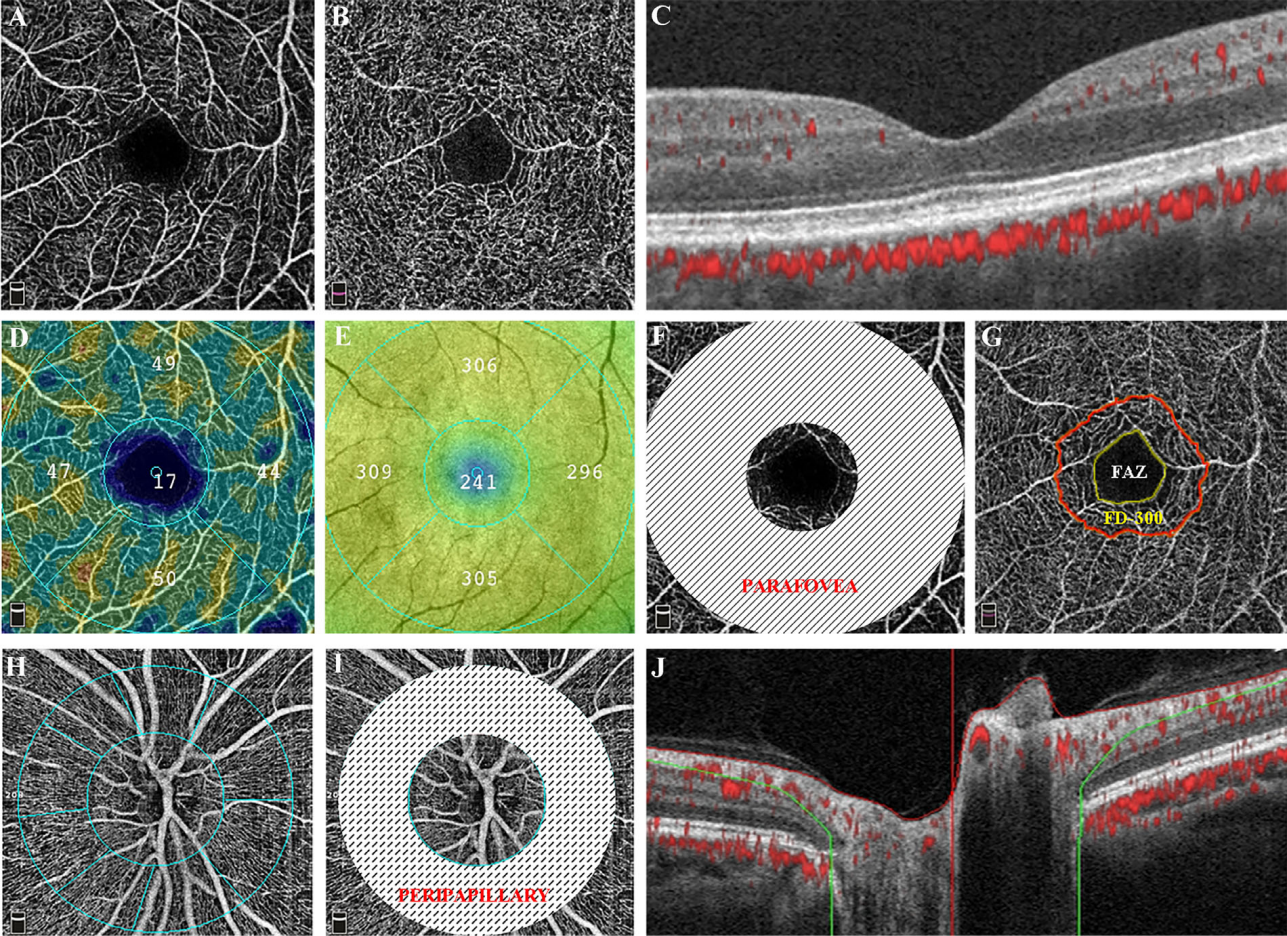
Representative OCTA images obtained by RTVue XR Avanti device (ReVue software, version 2017.1). (**A–G**) Macula was screened by angio retina modes (HD 3 × 3 mm). (**H–J**) Optic disc was scanned by angio disc (HD 4.5 × 4.5 mm). **A** OCTA image of SCP, defined as ILM to IPL -10 µm. **B** OCTA image of DCP, defined as IPL -10 µm to OPL +10 µm. **C** The corresponding macular structural OCT B-scan image. **D** Vessel density was automatically measured in SCP. **E** Thickness of the full retina was automatically calculated from ILM to RPE. **F** Macula was regionally measured in the whole ETDRS grid area, which comprised 2 concentric rings: 1 mm fovea center and 1 to -3 mm parafovea area. **G** The FAZ area was automatically detected on the Retina slab (ILM to OPL +10 µm) and FD-300 was determined as vessel density within 300 µm width ring surrounding the FAZ. **H** OCTA image of RPC, defined as ILM to the posterior border of RNFL. **I** Peripapillary grid was defined by 2 rings of 2 mm and 4 mm centered on disc center. **J** The corresponding peripapillary structural OCT B-scan image.

Ocular examinations included measurements of visual acuity (plus pin-hole acuity), tonometry, slit-lamp assisted biomicroscopy of the anterior segment, and ocular biometry consisting of the central corneal thickness, corneal curvature, anterior chamber depth, lens thickness, and axial length by applying optical low-coherence reflectometry (Lenstar 900 Optical Biometer; Haag-Streit, Koeniz, Switzerland). Pupils were dilated by applying 0.5% tropicamide and 0.5% phenylephrine hydrochloride eye drops. Two 45 degrees fundus photographs centered on the optic nerve head and macula were taken by a digital fundus camera (CR6-45NM; Cannon, Inc., Ōsta, Tokyo, Japan). Both optical coherence tomographic (OCT) and OCTA images were obtained using a commercially RTVue XR Avanti device (ReVue software, version 2017.1; Optovue Inc., Fremont, CA, USA). The OCTA algorithm and imaging processing technique have been described deeply in previous publications.^9^^–^^13^ The software performed automatic segmentation of retinal layers, and the vessel density was calculated as the percentage of areas occupied by detected vasculature within the selected regions and layers (Fig. 1). In addition, the built-in projection artifact removal (PAR) algorithm could remove the projection artifacts, as the more superficial flowing red blood cells could create fluctuating shadows in the deeper layers. For all study participants, angio retina mode (HD 3 × 3 mm; Figs. 1A–G) and angio disc mode (HD 4.5 × 4.5 mm; Figs. 1H–J) mode were performed. In the macular area, superficial capillary plexus (SCP; see Fig. 1A), which extends from the inner limiting membrane (ILM) to 10 µm above the inner plexiform layer (IPL) and the deep capillary plexus (DCP; see Fig. 1B), which is defined as 10 µm above the IPL to 10 µm beneath the outer plexiform layer (OPL) were analyzed. In addition, parafovea microvasculature was regionally measured in the whole ETDRS grid area, which comprised 2 concentric rings: 1 mm fovea center and 1 to 3 mm parafovea area (see Fig. 1F). Thickness of the full retina (from ILM to retinal pigment epithelium [RPE]; see Fig. 1E) and the geometrical FAZ (see Fig. 1G) measurements were also taken into analysis. The FAZ area was automatically detected on the retina slab (ILM to OPL + 10 µm). The FAZ Acircularity Index was defined as the ratio between the measured perimeter and the perimeter of the same size circular area, and FD-300 was determined as the vessel density within a 300 µm width ring surrounding the FAZ. In the disc area, the radial peripapillary capillaries (RPC) layer, which is defined as the ILM to the posterior border of retinal nerve fiber layer (RNFL), and the peripapillary RNFL thickness were regionally measured between the 2 rings of 2 mm and 4 mm centered on disc center (see Figs. 1H, 1J). Both fundus photographs and OCTA scans were screened and assessed by experienced and trained ophthalmologists (W.J.Z. and Q.W.). If any objection existed, the photographs were reassessed by a panel, including several ophthalmologists (W.J.Z., Q.W., Y.X.W., J.B.J., and W.B.W.). Both eyes were evaluated but only the data of right eyes were enrolled into analysis.

### Statistical Analysis

All statistical analyses were performed using a commercially available statistical software program (SPSS for Mac, version 25.0; IBM/SPSS, Chicago, IL, USA). Variables were presented as mean ± standard deviation or frequency and 95% confidential interval (CI) was calculated for continuous variables, and percentages for categorical variables. Univariate linear regression models were used to analyze potential associations between the ASCVD systemic risk factors and the OCTA parameters, assessing the strength of each association. Multivariate linear regression analyses were then performed, and a stepwise selection of the significant independent ASCVD variables (*P* < 0.1) and potential confounding factors were included in the model for retinal microvascular parameters. In all analyses, *P* < 0.05 was considered to have statistical significance.

## Results

A total of 390 participants went through the OCTA examination. Twelve applicants with a medical history of self-reported diabetes, or medication of hypoglycemic agents were considered to be diagnosed with DM, and five applicants reported previous ocular diseases or surgeries and were excluded from the study. After our grading process, 15 individuals were found with abnormalities on color fundus images and 5 applicants were excluded due to unreadable OCTA scans or fundus images. Herein, 353 eligible individuals (166 men, 47%) aged from 22 years to 82 years, mean age 49.86 ± 11.41 years, met our criteria and were recruited into our analyses. The descriptive statistics of systemic stressors are shown in Table 1, and the OCTA variables distributions are shown in Table 2.

**Table 1.** Frequency Distribution of Systemic Variables in the Participants of the Kailuan Eye Study

Variables	Mean^a^
Age, years	49.86 ± 11.41
Sex, male	166 (47%)
Visual acuity	0.61 ± 0.34
Intraocular pressure, mm Hg	15.50 ± 2.79
Axial length, mm	23.90 ± 1.26
Body mass index, kg/m^2^	24.27 ± 3.23
Waist-hip ratio	0.90 ± 0.07
Smoking history	71 (20.1%)
Systolic blood pressure, mm Hg	128.12 ± 18.76
Diastolic blood pressure, mm Hg	79.37 ± 10.58
MABP, mm Hg	95.62 ± 12.20
Heart rate, beats / minute	71.48 ± 9.78
FPG, mmol/L	5.32 ± 1.19
Creatinine, µmoI/L	66.07 ± 34.43
Uric acid, µmol/L	315.09 ± 82.54
HDL-C, mmol/L	1.62 ± 0.62
LDL-C, mmol/L	2.90 ± 0.83
Triglyceride, mmol/L	1.71 ± 2.54
Total cholesterol, mmol/L	5.26 ± 3.00
Total bilirubin, µmol/L	14.54 ± 5.22
GPT, U/L	18.85 ± 11.69
H-CRP, mg/L	2.13 ± 2.92

a All data were listed as mean ± standard deviation or frequency (percent).

**Table 2.** Descriptive Statistics of OCTA Parameters (Retina 3 × 3 mm, Disc 4.5 × 4.5 mm) in the Kailuan Eye Study

Variables	Mean ± Standard Deviation
SCP density, %: whole ETDRS grid	45.63 ± 3.84
Fovea	15.02 ± 5.90
Para fovea	48.4 ± 4.10
DCP density, %: whole ETDRS grid	50.21 ± 3.25
Fovea	29.05 ± 7.52
Para Fovea	52.73 ± 3.30
Full retina thickness, µm	311.6 ± 16.19
FAZ area, mm^2^	0.33 ± 0.13
FAZ perimeter, mm	2.29 ± 0.46
FAZ Acircularity Index	1.14 ± 0.04
FD-300 area density, %	49.3 ± 4.17
Peripapillary RNFL thickness, µm	113.76 ± 14.08
RPC density, %	52.47 ± 3.66
Scan Quality Index	8.08 ± 0.99

**Figure 2.**
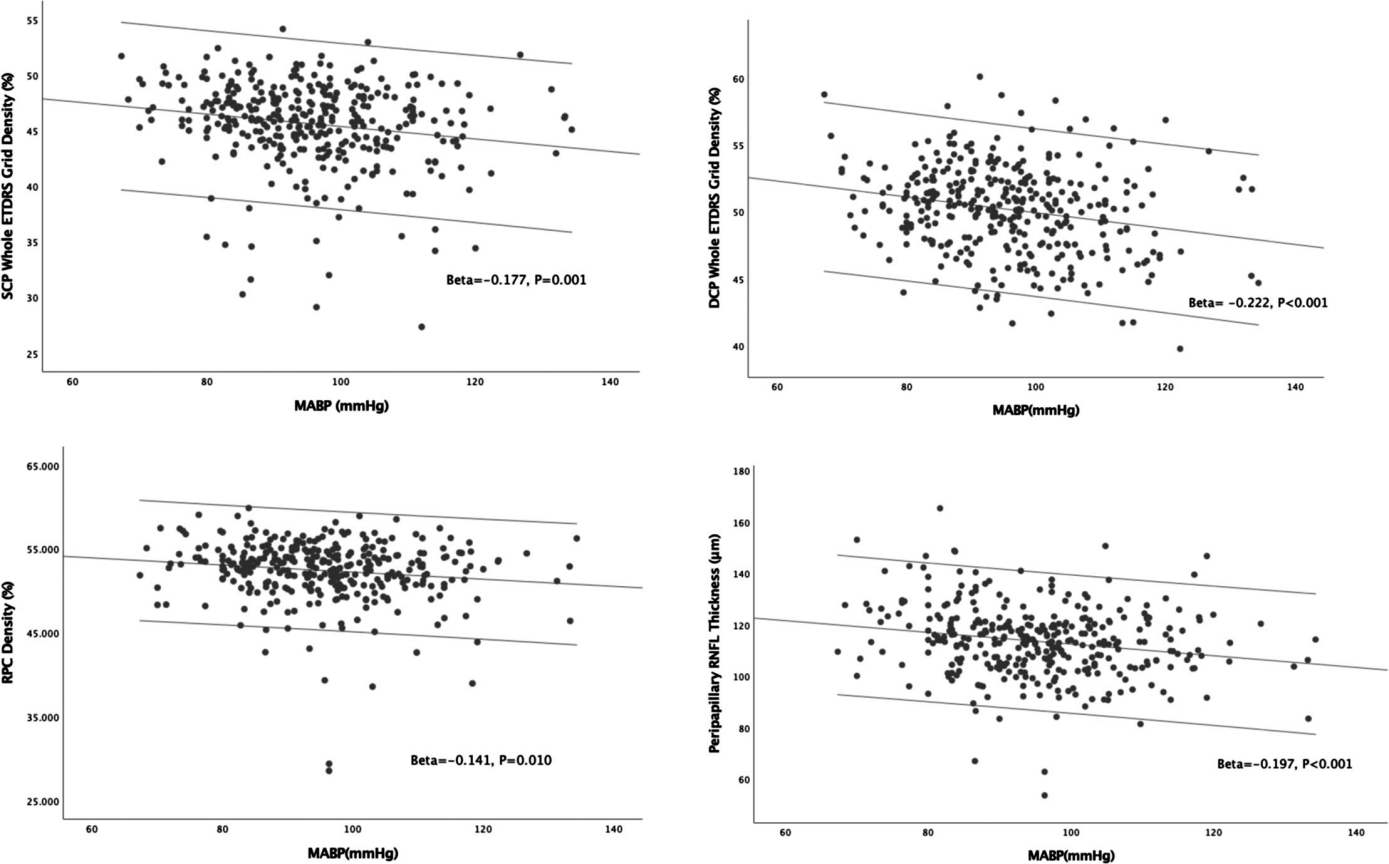
Scatter plots showing relationships between MABP and OCTA parameters. *Solid lines* inside the plots represent the univariate linear models.

**Figure 3.**
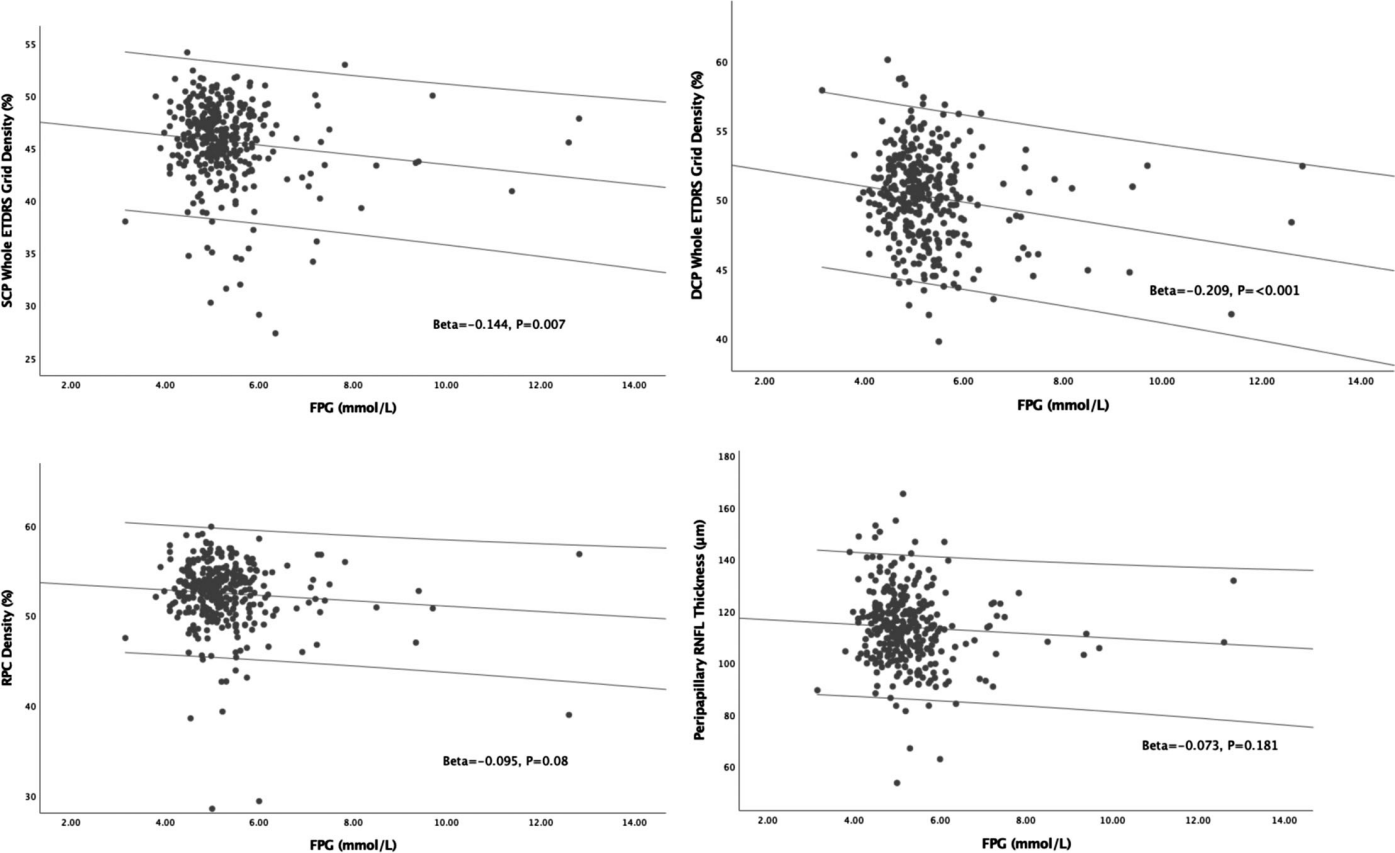
Scatter plots showing relationships between FPG and OCTA parameters. *Solid lines* inside the plots represent the univariate linear models. There were no correlations found between FPG and RPC density and peripapillary RNFL thickness (*P* = 0.08 and *P* = 0.181).

In univariate linear regression models, MABP (Fig. 2) was found to be significantly correlated to SCP density (*P* = 0.001), DCP density (*P* < 0.001), RPC density (*P* = 0.01), and peripapillary RNFL thickness (*P* < 0.001). As shown in Figure 3, FPG was found to be significantly correlated to SCP density (*P* = 0.007) and DCP density (*P* < 0.001). The relationships between other systemic stressors and OCTA parameters can be found in Supplementary Table S1.

**Figure 4.**
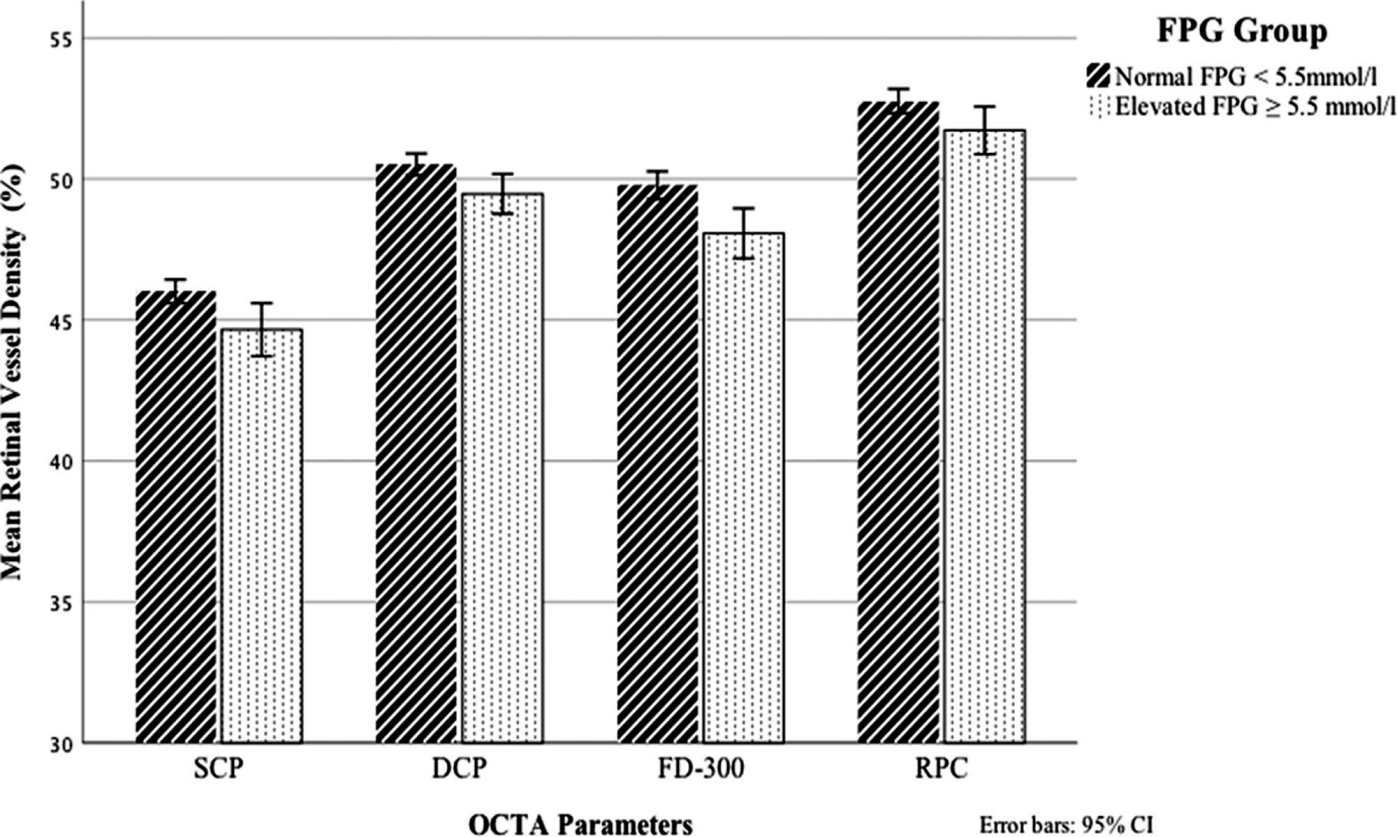
The distribution of OCTA mean vessel density in FPG normal (*N* = 253, mean FPG = 4.88 ± 0.37 mmol/L) and elevated (*N* = 100, mean FPG = 6.44 ± 1.71 mmol/L) groups stratified by the American Diabetes Association (ADA) criteria (FPG cutoff value 5.5 mmol/L). The mean retinal vessel density of SCP, DCP, FD-300, and RGC in normal FPG group were significantly higher than in the elevated FPG group (*P* = 0.003, *P* = 0.006, *P* = 0.019, and *P* < 0.001 respectively).

To generally describe the retinal vessel density altered by FPG levels, we stratified our study cohort into two groups following the American Diabetes Association (ADA) criteria, in which the cutoff FPG value was defined as 5.5 mmol/L. The vessel density of SCP, DCP, FD-300, and retinal ganglion cell (RGC) in the normal FPG group were significantly higher than in the elevated FPG group (*P* = 0.003, *P* = 0.006, *P* = 0.019, and *P* < 0.001, respectively; Fig. 4).

Furthermore, we performed multivariable linear regression analyses for each OCTA variable in which we put all significant systemic variables (*P* < 0.1) and potential confounding factors from the univariate linear regression models. As shown in Table 3, decreased SCP density was significantly correlated with elder age (*P* < 0.001) and male gender (*P* = 0.002). Lower DCP density was associated with elder age (*P* = 0.001), male gender (*P* < 0.001), and higher FPG (*P* = 0.008; beta = −0.134; B = −0.366; VIF = 1.088; 95% CI = −0.635 to −0.096). In the RPC model, male gender (*P* < 0.001), longer axial length (*P* < 0.001; beta = −0.274; B = −0.788; VIF = 1.024; 95% CI = −1.078 to −0.499), and higher FPG (*P* = 0.029; beta = −0.112; B = −0.355; VIF = 1.015; 95% CI = −0.673 to −0.037) were significantly associated with reduced RPC density. A negative trend between peripapillary RNFL thickness was found with age (*P* < 0.001), male gender (*P* = 0.005), and axial length (*P* < 0.001; beta = −0.238; B = −2.635; VIF = 1.171; 95% CI = −3.849 to −1.422).

**Table 3.** Multivariable Linear Regression Analysis between OCTA Parameters and Systemic Stressors in the Kailuan Eye Study

	SCP Density		DCP Density		RPC Density		RNFL Thickness	
Parameters	*P* Value	Standardized Beta	*P* Value	Standardized Beta	*P* Value	Standardized Beta	*P* Value	Standardized Beta
FPG, mmol/L			**0.008**	−0.134	**0.029**	−0.112		
Age, years	**< 0.001**	−0.293	**0.001**	−0.173			**< 0.001**	−0.206
Sex, male	**0.002**	−0.159	**< 0.001**	−0.317	**< 0.001**	−0.219	**0.005**	−0.152
Axial length, mm					**< 0.001**	−0.274	**< 0.001**	−0.238

The figures in boldface represent statistical significance.

Additionally, multivariable linear regression model was made between FPG and ocular parameters adjusting the confounding systemic factors. A higher FPG concentration was significantly correlated with lower DCP density (*P* = 0.006; beta = −0.153; B = −0.056; VIF = 1.210; 95% CI = −0.096 to −0.017), and higher intraocular pressure (*P* = 0.006; beta = 0.141; B = 0.060; VIF = 1.033; 95% CI = 0.018 to 0.103), after adjusting MABP (*P* = 0.001; VIF = 1.438) and sex (*P* = 0.042; VIF = 1.342; Table 4).

**Table 4.** Multivariable Linear Regression Analysis between FPG and Systemic Factors in the Kailuan Eye Study

		Standardized	Unstandardized	95% Confidence	Variance Inflation
Parameters	*P* Value	Coefficients Beta	Coefficients B	Interval for B	Factor
MABP, mm Hg	**0.001**	0.208	0.020	0.009 to 0.032	1.438
Age, years	**0.002**	0.178	0.019	0.007 to 0.031	1.314
Intra ocular pressure, mm Hg	**0.006**	0.141	0.060	0.018 to 0.103	1.033
DCP Density, %	**0.006**	−0.153	−0.056	−0.096 to −0.017	1.210
Sex, male	**0.042**	−0.118	−0.283	−0.555 to −0.011	1.342

The figures in boldface represent statistical significance.

## Discussion

ASCVD has been one of the leading causes of death globally, especially in patients with diabetes. As retinal vessels share similar vascular magnitude and pathological changes with coronary vessels,^17^ retinal microvascular conditions are of great value to represent the current systemic or coronary vasculature status. Investigating how ASCVD risk factors contribute to the retinal alterations is also an essential component for preventative interventions. To our knowledge, few studies have addressed the correlations between comprehensive systemic risk factors and retinal microvascular measurements in a generally healthy Chinese population.

Interestingly, although several main ASCVD variables were strongly correlated with OCTA parameters in the univariate linear regressions, only FPG was found to have statistical significance in multivariate models. Persistent hyperglycemia can result in vascular endothelial cell dysfunctions and increase the risks of microvascular complications. The progression from impaired fasting glucose (IFG) or IGT to diabetes can take years, thus individuals with rising glycemic concentration are possibly suffering from early metabolic and vascular abnormalities without having been diagnosed with diabetes.^18^^,^^19^ Previous studies on the blood flow changes of retinal vessels in diabetic eyes were controversial,^20^^,^^21^ yet decreased capillary density was consistently reported in both superficial and deep plexus in non-DR (NDR) eyes compared with healthy controls.^22^^–^^24^ Our recent diabetic eye study reported that microvascular retinal abnormalities were detected in 40.4% of patients with NDR T2DM by OCTA, including microaneurysms and capillary dropouts in both plexuses.^25^

Our present study suggests the FPG concentration is independently correlated with retinal microvasculature in nonretinopathy individuals (Table 3), which could be a prior self-regulating compensation by lowering microvascular density due to hyperglycemia. The vascular abnormalities caused by chronic glucose excess, such as increase of protein kinase C and oxidative stress, might already be present in undiagnosed diabetes and followed by serial biochemical changes, and result in capillary dropouts.^26^ Accordingly, the capillary loss could leave nonperfusion areas in hypoxic exposure.^27^ Ischemic retina could increase the expression of VEGF,^28^ so, hypothetically, elevated VEGF may arise at an early stage, and trigger the process of vascular lesions.

Furthermore, the FPG multivariable linear model (Table 4) confirmed that the DCP was the only concomitant OCTA parameter. The variation of the FPG strength within different layers and zones is worth investigating as well. Our results agreed with previous DR research. Kaizu's^29^ group discovered that early-staged DR had significantly decreased DCP density only, and the DCP/SCP density ratio in NDR was higher than DR eyes. Chen and colleagues^30^ introduced the fractal dimensional parameter, which was specifically associated with DCP in NDR. Considering all our study applicants are NDR, we may presume that the deep layer could be the onset of the diabetic retinal microvasculature changes. The morphology difference between the two capillary plexuses may substantially support our hypothesis. The whole retina is supplied by two different arteries, the inner retina (ILM-INL) by central retinal artery and the outer retina (OPL-RPE) by short posterior ciliary artery. According to Scholl's group,^31^ the FAZ generates a watershed between the different retinal circulation and therefore could reduce the extravascular fluid resorption in the parafovea area. Meanwhile, the radially oriented Henle fiber layer, which lies in the OPL (DCP), inclines to the accumulation of extravascular fluid due to its morphologically loose structure. Our data also agreed with this anatomic feature that the deep plexus had denser capillaries than the superficial plexus (Table 2). Furthermore, it is well-known that leukostasis plays an important role in diabetic vascular abnormalities. According to Spaide's theory,^32^ leukocytes tend to plug very small vessels, and, due to its different geometry, it is more likely to occur in the DCP. Hence, the DCP is more prone to be affected because of the occlusion of these vessels.

Additionally, the correlation specifically in the DCP might mirror the mechanism of cystoid macular edema (CME). Chronic hyperglycemia could damage the blood-retinal barrier, and eventually result in diabetic macular edema (DME), which is the leading cause of blindness in diabetes.^33^ On the histological level, CME presents extra fluid accumulated in the OPL and INL (DCP), with Müller cells swelling.^32^ Previous OCTA studies have reported a decreased density or nonperfusion area in the cystoid spaces and more microaneurysms are detected in the DCP compared to the SCP.^11^^,^^32^^,^^34^ This particular discrepancy of the two capillary plexuses changes might also give evidence to the existence of a bulk interstitial flow management from superficial to the deep plexus by the ocular glymphatic system. The fluid circulation system was first described in the brain by Iliff,^35^ and recently scientist have found substantial evidence that this paravascular transport system exists in the retina.^36^ In the CME, the decreased or dropout areas in the DCP would disrupt the normal fluid transportation as mediated by Müller cells, leaving lesions in the deep plexus.^32^ Overall, understanding the vulnerability of the DCP may lead to new therapeutic strategies of DR.

The relationship concerning RPC density with FPG remains unclear. Previous studies have documented RPC and RNFL changes in NDR eyes.^37^^,^^38^ In our univariate analysis, FPG indicated a secondary impact (*P* = 0.08; see Fig. 3) on RPC, and an independent impact (*P* = 0.029) in the multivariable linear model but was removed in the independent FPG model. Hence, we may suppose that FPG's strength on RPC is relatively secondary than macular capillary, but future study is needed to support this hypothesis.

Additionally, intraocular pressure was found positively associated with the FPG level. The outcome agreed with previous publications that hyperglycemia could increase proliferation in trabecular meshwork cells and generate abnormal aqueous humor circulation.^39^ Although all the participants had normal ocular pressure when examined, this trend could also suggest the adverse impact of glucose on ocular fluid transportation in healthy people.

Furthermore, we found both age and sex contributed to a huge impact on retinal microvasculature after adjusting all related systemic or ocular variables. As expected from former studies,^40^^,^^41^ age was inversely correlated with macula capillary density and RNFL thickness. Previous investigations demonstrated that biochemical changes happen to blood vessels with age, which could lead to abnormal functions and morphology in the brain vasculature,^42^ and thus may result in hypoperfusion in retinal vasculature. In addition, male patients were associated with reduced retinal capillary density and thinner RNFL thickness, which was in concert with previous OCTA studies in different ethnicities.^40^^,^^41^^,^^43^ The reason why men had lower vessel densities remains unclear. Yet from our study cohort, men had higher rates of smoking history and worse ASCVD risk factors, which could possibly illustrate the issue. There is also a possibility that women had more medical self-awareness or accuracy of surveys, indicating potentially more precise self-reported medical history or even less undiagnosed diabetes.

We did not find any independent relationships to other main ASCVD risk factors. Our results agreed with previous large-scaled epidemic studies, where there were no linear relationships discovered between capillary densities and hypertension.^40^^,^^41^ However, MABP, LDL-C, and smoking history showed strong associations with OCTA parameters in the univariate linear analyses, which implies the strength of these variables should still be considered when analyzing OCTA, isolating the effects of these determinants is inappropriate when several stressors could occur together.

Nevertheless, our study has several limitations. First, because the majority of our study cohort were nondiabetic, we did not collect the HbA1c parameter routinely, which better describes the average level of glucose for the past few months. Some studies reported that the HbA1c level was not correlated with retinal capillary density in diabetic eyes,^13^^,^^20^^,^^44^ whereas some proposed negative associations with SCP in children with T1D NDR.^45^ After all, the relationships we discovered between diabetic parameter with retinal microvasculature may vary if concerning HbA1c rather than FPG. Second, the study cohort was not recruited as population-based, and miners tend to have unhealthier lifestyles, lower educations, and less common medical knowledge, therefore the percentage of undiagnosed diabetes could be above average and may influence the distributions of OCTA parameters. Third, both the two 45 degrees fundus photographs and OCTA scans were center limited, so lesions developed in the peripheral area were possibly left undetected. Finally, because our present study is cross-sectional, the correlations we found may not be eligibly defined as causal relationships. After all, further large-scaled perspective studies are required for supporting the hypothesis and evidence of correlations between ASCVD factors and retinal microvasculature.

Despite the limitations outlined above, our study also has strengths. To minimize the statistic disturbance, we only enrolled data of each applicant's right eye into analysis. Based on the various ASCVD data and questionnaires obtained from each follow-up in a 10-year period, we had more comprehensive information of each participant's physical conditions. Both macular capillary plexuses (SCP/DCP) and FD-300 density were included in the study as the measurements of the macula margin were different and therefore we could thoroughly describe the macular microvascular perfusion alterations.^13^ Moreover, the axial length parameter was adjusted in the multivariable analyses, which has been proved to alter the retinal vasculature significantly.^15^^,^^41^ Consequently, we were able to include comprehensive potential confounders in adjusted multivariate models and explore the independent risk factors thoroughly. More importantly, the study cohort is focused on people without history of diabetes or ASCVDs and with clean color fundus photographs, so the results could well describe OCTA's ability to detect early-stage alterations.

In conclusion, diabetes is a chronic vascular disease and accompanies ASCVD due to shared risk factors. Our present study indicated that DCP density inversely correlated with FPG concentration in people without diabetes. These data not only suggest hyperglycemia can cause early retinal capillary alterations in patients with no clinical signs of retinopathy yet, but also indicate that OCTA could detect regional subclinical changes, and routine OCTA screening may be beneficial for preventions, grading, and managements of patients with high risk of ASCVD.

## Supplementary Material

Supplement 1
