# Supplementary material for: Systemic Stressors and Retinal Microvascular Alterations in People Without Diabetes: The Kailuan Eye Study
Source: Invest Ophthalmol Vis Sci. 2021 Feb 17;62(2):20. doi: 10.1167/iovs.62.2.20 (PMC7900855; doi:10.1167/iovs.62.2.20)
Supplement: Supplement 1 [file iovs-62-2-20_s001.pdf]

Supplementary materials

TABLE S1. Univariate Linear Regression of Systemic Variables with OCTA Parameters in the Kailuan Eye Study

	SCP Density		DCP Density		RPC Density		RNFL Thickness	
Variables	Beta[a]	P-Value	Beta[a]	P-Value	Beta[a]	P-Value	Beta[a]	P-Value
Age (years)	-0.331	**<0.001**[b]	-0.288	**<0.001**[b]	-0.011	0.835	-0.165	**0.002**[b]
Sex (Male)	-0.230	**<0.001**[b]	-0.368	**<0.001**[b]	-0.254	**<0.001**[b]	-0.228	**<0.001**[b]
Visual Acuity	-0.052	0.329	-0.082	0.124	0.247	**<0.001**[b]	0.078	0.155
Intra Ocular Pressure (mmHg)	-0.059	0.267	-0.026	0.633	-0.102	0.061	-0.072	0.188
Axial Length (mm)	0.084	0.115	0.091	0.088	-0.286	**<0.001**[b]	-0.185	**0.001**[b]
Body Mass Index (kg/m^2)	-0.166	**0.002**[b]	-0.011	0.842	-0.091	0.095	-0.090	0.098
Waist-Hip Ratio	-0.039	0.479	-0.047	0.395	0.021	0.711	0.004	0.947
Smoking History	-0.168	**0.002**[b]	-0.314	**<0.001**[b]	-0.098	0.070	-0.082	0.133
Systolic Blood Pressure (mmHg)	-0.205	**<0.001**[b]	-0.184	**0.001**[b]	-0.082	0.133	-0.161	**0.003**[b]
Diastolic Blood Pressure (mmHg)	-0.125	**0.019**[b]	-0.220	**<0.001**[b]	-0.170	**0.002**[b]	-0.198	**<0.001**[b]
MABP (mmHg)	-0.177	**0.001**[b]	-0.222	**<0.001**[b]	-0.141	**0.010**[b]	-0.197	**<0.001**[b]
Heart Rate (beats / minute)	-0.011	0.843	-0.061	0.271	0.016	0.782	0.016	0.772
FPG (mmol/L)	-0.144	**0.007**[b]	-0.209	**<0.001**[b]	-0.095	0.080	-0.073	0.181
Creatinine （μmol/L）	-0.056	0.296	-0.146	**0.006**[b]	-0.067	0.215	-0.089	0.102
Uric Acid (μmol/L)	-0.042	0.428	-0.063	0.239	-0.207	**<0.001**[b]	-0.161	**0.003**[b]
HDL-C (mmol/L)	0.020	0.706	0.040	0.459	0.082	0.130	0.048	0.379
LDL-C (mmol/L)	-0.129	**0.015**[b]	-0.123	**0.021**[b]	-0.028	0.602	-0.070	0.196
Triglyceride (mmol/L)	-0.031	0.556	-0.093	0.082	-0.050	0.355	-0.058	0.286
Total Cholesterol (mmol/L)	0.016	0.761	0.039	0.469	0.051	0.347	-0.028	0.607
Total Bilirubin (μmol/L)	-0.002	0.976	0.089	0.095	-0.088	0.106	-0.083	0.128
GPT (U/L)	-0.051	0.337	-0.155	**0.004**[b]	-0.148	**0.006**[b]	-0.137	**0.012**[b]
H-CRP (mg/L)	-0.107	**0.046**[b]	-0.005	0.920	-0.021	0.708	-0.049	0.372

a Standardized Coefficients Beta and P-values were calculated from univariate linear regression with OCTA Parameters.
b P ≤ 0.05 considered significant.
